# What Could Computerized Brain Training Learn from Evidence-Based Medicine?

**DOI:** 10.1371/journal.pmed.1001758

**Published:** 2014-11-18

**Authors:** Druin Burch

**Affiliations:** Public Library of Science, Cambridge, United Kingdom; PLoS Medicine, United States of America

## Abstract

In a Perspective linked to the study by Michael Valenzuela and colleagues, Druin Burch considers the context, content, and limitations of what we know about computerized cognitive enhancement.

*Please see later in the article for the Editors' Summary*

**Linked Research Article**

This Perspective discusses the following new study published in *PLOS Medicine*:

Lampit A, Hallock H, Valenzuela M (2014) Computerized Cognitive Training in Cognitively Healthy Older Adults: A Systematic Review and Meta-Analysis of Effect Modifiers. PLoS Med 11(11): e1001756. doi:10.1371/journal.pmed.1001756

Michael Valenzuela and colleagues systematically review and meta-analyze the evidence that computerized cognitive training improves cognitive skills in older adults with normal cognition.

Computerized cognitive training (CCT) “is modestly effective at improving cognitive performance in healthy older adults”, find Michael Valenzuela and colleagues, who systematically review the evidence in this week's *PLOS Medicine* [1]. This is a conclusion of value to academics in the field and to those with interest in selling training programmes. The value to others depends on how well they understand the conclusion's limits.

CCT has a market approaching a billion dollars a year [2] and an uncertain evidence base. One company's website offers “…a brain training system built and tested by an international team of more than 100 top neuroscientists”, for helping “your brain to make real improvements” [3]. Does the new review support this conception of computer programmes, which in the past would have been described as games or exercises? Either these companies have discovered a novel means of enhancing cognitive performance, or these programs are no more effective—but considerably more expensive—than Sudoku and crosswords [4].

Adherents to evidence-based medicine seek reliable proof that interventions make a helping difference, and they attempt to make sure that evidence supports branding. Outside of medicine, consumers are treated as capable of making up their own minds about purchases with less help from regulatory oversight. The participants in the studies reviewed here were not enrolled as patients, and most users of CCT programmes have not been prescribed them by physicians. Being asked to pay money for “standardized computerized tasks with clear cognitive rationale” [1], a fuller description of CCT given by Valenzuela and colleagues, offers a cooler prospect than that implied by “a faster, sharper brain” [3].

People driven by fear of cognitive decline might reasonably seek guidance about CCT programmes from their physicians. It is otherwise unclear whether providing advice about CCT to those without related symptoms is the business of doctors, except perhaps in the context of the WHO's famously all-embracing definition of health as a “state of complete physical, mental, and social well-being” [5]. Medicine has, however, developed reliable techniques for determining the effects of complex interventions when those interventions cannot be soundly tested by intuition, observation, and argument. Medicine may have no right to say whether people should use CCT, with what ardour, or in what circumstances, but it is well placed to say how CCT should be tested to see what effects it delivers.

Practising Sudoku may have benefits [4], but not necessarily in terms of increasing your capacity for foreign languages, driving, drawing, or even crosswords. Doing something repeatedly can make you better at it, which is not the same as saying it makes you better. For that reason, Valenzuela and colleagues' review is of studies assessing how practice at particular tasks transferred to more general ones. They estimate that improvements from group-based CCT “may approximate an average relative improvement of 1 point” on the Mini-Mental State Examination [1]. No outcomes, though, were based on differences in actual activities of living. In line with the evidence, the review was also limited to assessing “change in performance from baseline to immediately post-training”. It could not evaluate whether any of the small changes detected (which may or may not extrapolate to settings outside of specific cognitive tests) persist, even to the next day.

Valenzuela and colleagues show effects that are statistically significant but uncertain in their impact on human capacity and performance. Their review, they note, “provides no indication about the durability of the observed gains, nor their transfer into real-life outcomes such as independence, quality of life, daily functioning, or risk of long-term cognitive morbidity”. Such a careful summary is worthwhile, as much for clarifying what is not yet known as for what is. It suggests that clinical trial methodology remains necessary to examine the proposed benefits of CCT. As bloodletting for infection was accepted as beneficial less than a century ago [6], evidence rather than belief should serve as the basis for decision making about interventions [7],[8]. It is required here.

The review's message regarding the limitations of CCT may fail to reach many who are lured by the promises prominent on training websites. Consumers may not make informed decisions, and those experiencing symptoms of dementia or the fear of such symptoms may mistake CCT products for proven medical interventions. That care needs to be taken over the potential for such misunderstanding is plain. Even clinical situations that involve informed consent may not result in realistic expectations: in a study of patients undergoing coronary angioplasty for stable angina between 2009 and 2011, when the evidence showed it did not prevent death, 90% who consented agreed to the procedure in the belief that it did [9]. Moving closer to CCT, the “Mozart effect” showed how experiments on intelligence [10] could be rapidly and influentially misinterpreted, leading to the widespread sale and purchase of what were thought to be proven tools for useful cognitive enhancement but which were not [11].

If cardiologists and patients together cannot interpret clear, high-quality evidence, it is reasonable to worry how a limited evidence base will collide with a public wishing to believe in it and a billion-dollar industry based on encouraging them. The benefits and limitations of CCT need to be clearly demonstrated and communicated if the public are not to invest money and time with unrealistic expectations of what they will gain. For CCT to promise more than it delivers and for it to encourage people to oversimplify such a serious matter as the care of their own minds would be a grave pity. Those doctors who once used animal testicles to refresh human vitality and sexual potency, and to augment intelligence and reverse its age-related decline, made a great deal of money from their work. They also performed it sincerely, showed it to be supported by clear rationale, and demonstrated that it made encouraging changes to short-term outcomes [12]–[14]. The allure of CCT is real but so are the potential harms of mistaking and overestimating what is currently sold in its name.

CCT programmes may or may not be medicine, but to be properly assessed their powers certainly require the tools of evidence-based medicine. Does a billion-dollar gap exist between our knowledge about “standardized computerized tasks with clear cognitive rationale” and the industry selling them? Valenzuela and colleagues' overview of the evidence for CCT in cognitively intact older adults suggests it does. It makes clear what remains to be discovered and suggests promising lines of inquiry. Their paper is of use to those planning thoughtful research in the field. It will not be the fault of the authors if the small effects shown, specific to particular subgroups and short-term outcomes, lead to the marketing departments of profitable companies declaring, with added confidence and effect, that their products are “scientifically proven” and doing so without being legally responsible for consumers or patients mistaking what that means.
